# Comprehensive kinome NGS targeted expression profiling by KING-REX

**DOI:** 10.1186/s12864-019-5676-3

**Published:** 2019-04-23

**Authors:** Giovanni Carapezza, Carlo Cusi, Ettore Rizzo, Laura Raddrizzani, Sebastiano Di Bella, Alessio Somaschini, Antonella Leone, Rosita Lupi, Margherita Mutarelli, Vincenzo Nigro, Diego di Bernardo, Paolo Magni, Antonella Isacchi, Roberta Bosotti

**Affiliations:** 1 NMS Oncology, Nerviano Medical Sciences Srl, Nerviano, MI Italy; 2 Department of Electrical, Computer, and Biomedical Engineering, University of Pavia, Pavia, Italy; 3 enGenome s.r.l., Pavia, Italy; 4 Telethon Institute of Genetics and Medicine (TIGEM), Pozzuoli, NA Italy; 5 Medical Genetics, Department of Biochemistry, Biophysics and General Pathology, University of Campania ‘Luigi Vanvitelli’, Naples, Italy; 6 Department of Chemical, Materials and Industrial Production Engineering, University of Naples Federico II, Naples, Italy

**Keywords:** Kinome gene expression, NGS RNA targeted panel

## Abstract

**Background:**

Protein kinases are enzymes controlling different cellular functions. Genetic alterations often result in kinase dysregulation, making kinases a very attractive class of druggable targets in several human diseases. Existing approved drugs still target a very limited portion of the human ‘kinome’, demanding a broader functional knowledge of individual and co-expressed kinase patterns in physiologic and pathologic settings. The development of novel rapid and cost-effective methods for kinome screening is therefore highly desirable, potentially leading to the identification of novel kinase drug targets.

**Results:**

In this work, we describe the development of KING-REX (KINase Gene RNA EXpression), a comprehensive kinome RNA targeted custom assay-based panel designed for Next Generation Sequencing analysis, coupled with a dedicated data analysis pipeline. We have conceived KING-REX for the gene expression analysis of 512 human kinases; for 319 kinases, paired assays and custom analysis pipeline features allow the evaluation of 3′- and 5′-end transcript imbalances as readout for the prediction of gene rearrangements. Validation tests on cell line models harboring known gene fusions demonstrated a comparable accuracy of KING-REX gene expression assessment as in whole transcriptome analyses, together with a robust detection of transcript portion imbalances in rearranged kinases, even in complex RNA mixtures or in degraded RNA.

**Conclusions:**

These results support the use of KING-REX as a rapid and cost effective kinome investigation tool in the field of kinase target identification for applications in cancer biology and other human diseases.

**Electronic supplementary material:**

The online version of this article (10.1186/s12864-019-5676-3) contains supplementary material, which is available to authorized users.

## Background

Protein kinases constitute one of the largest families of enzymes that share a highly homologous catalytic domain (the kinase domain), which transfers the gamma phosphate from nucleoside triphosphates (ATP) to protein substrates, activating signal cascades and regulating multiple complex cellular processes. In several human diseases, such as cancer, kinases are often deregulated by gene alterations, leading to their anomalous expression and activation [1]. ‘Druggability’ by small molecule inhibitors, binding the conserved ATP-pocket, makes kinases therapeutically very attractive [2]: more than 500 kinases (the “kinome”) are encoded in the human genome, and kinase inhibitors now account for a quarter of all current drug discovery research and development efforts [3–6]. The clinical success of tyrosine kinase inhibitors is proven by a number of examples, such as imatinib in BCR–ABL1 fusion-positive leukaemia patients [7], or the more recent crizotinib and ceritinib in patients with lung carcinomas and mesenchymal tumors harboring anaplastic lymphoma kinase (ALK) fusions [8, 9]. Approved drugs, though, target a very limited portion of the human kinome, leaving much of the kinase therapeutic potential unexplored.

In cancer, besides the investigation of individual kinase genetic alterations [1], ‘kinomics’ approaches are emerging in the definition of co-expressed kinase functional roles in health and disease [10], as well as in integrative ‘polypharmacology’ approaches exploring synergizing effects of highly promiscuous kinase inhibitors [11]. Based on these considerations, the quest for novel kinase targets effective in oncogene-defined tumor types [5] is strongly encouraged to investigate tumor biology and to identify new candidate targets in specific disease contexts, also through the continuous generation of molecular data and the development of novel methods for kinome screening.

While ‘omics’ analysis approaches produce huge amounts of molecular information, requiring substantial computational power for data storage and management, next-generation sequencing (NGS) targeted RNA approaches enable the analysis of focused portions of the transcriptome. Currently, several companies supplying NGS solutions offer custom based RNA assays targeting gene transcripts, isoforms, splice junctions, noncoding RNAs, mutations and expressed fusion genes, with advantages over whole transcriptome sequencing in terms of reduced costs and simplicity of execution. Indeed, a number of commercial and custom approaches have been developed targeting small kinase panels (Illumina TruSight RNA Pan-Cancer panel [12], Illumina TruSight RNA Fusion Panel [13], Archer® FusionPlex® NGS assays [14–17]), allowing the detection of specific kinase gene and isoform expression, mutation and gene fusion events. However, to our knowledge, none of the reported NGS targeted RNA applications allow a comprehensive whole kinome expression analysis.

In this work, we describe the development of KING-REX (KINase Gene RNA EXpression), a kinome RNA targeted custom panel based on the TruSeq Targeted RNA expression Illumina kit (TREx, [18]) and coupled with a custom dedicated bioinformatics pipeline. We have conceived KING-REX as a solution for human kinome gene expression analysis on small/medium scale Illumina sequencers, requiring reduced computational resources in terms of storage space and data processing, with an additional feature in the analysis pipeline allowing the evaluation of 3′- and 5′-end transcript imbalances as a readout for the prediction of gene rearrangements.

## Results

### Design of a targeted RNA panel for the profiling of human kinome gene expression

Next Generation Sequencing (NGS) technologies currently offer the possibility to design panels of custom based assays for user defined sequences of interest. The focus of our work was the custom set-up of a targeted RNA procedure for a comprehensive gene expression analysis of the entire human kinome, intended for small/medium scale sequencers. Based on the Illumina TruSeq Targeted RNA Expression (TREx) approach [18], enabling a custom definition of up to 1000 assay panels, we assembled the KING-REX (KINome Gene RNA EXpression) panel, by selecting pre-designed assays with a specific targeting strategy, to combine the maximum capacity of the custom panel composition with the highest possible kinome coverage.

**Fig. 1:**
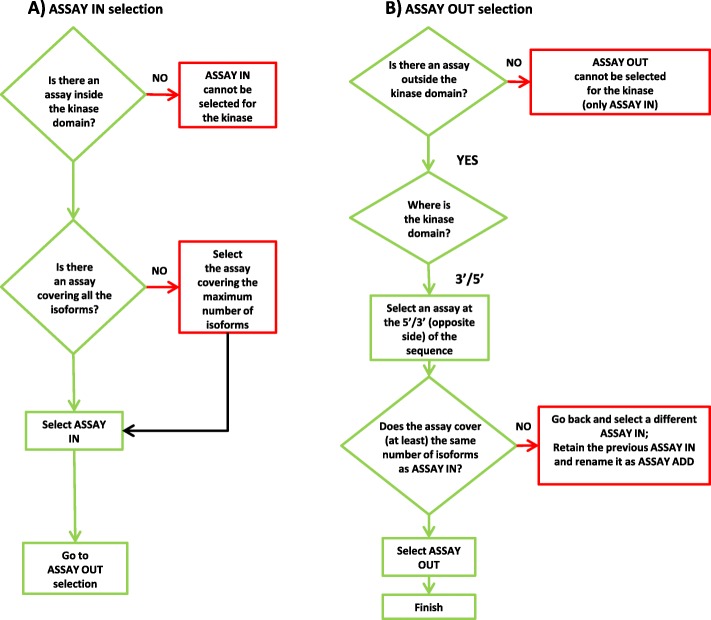
Flowchart of kinome assay selection. **a** Selection process of ASSAY IN, targeting the kinase domain; **b** Selection process of ASSAY OUT, outside the kinase domain; definition of ASSAY ADD upon re-selection of ASSAY IN. All the selected pre-designed assays were derived from Illumina DesignStudio Custom Assay Design Tool for use with the TruSeq Targeted RNA Expression (TREx) kit (Illumina, San Diego, CA, USA)

We started from compiling a comprehensive list of human protein kinases by integrating information from the currently available kinase resources [6, 19–23]. For 514 unique genes, clearly annotated as protein kinases, we retrieved genomic coordinates for 2230 kinase isoforms from the UCSC database, providing an acceptable confidence level for transcript annotation [24]. Kinase domain coordinates were then obtained for 1716 protein kinase isoforms harboring the catalytic domain, as reported in the Superfamily database ([25], superfamily ID number 56112, containing the ‘Protein kinases, catalytic subunit’ subfamily of interest), and directly mapped onto UCSC transcripts. We visualized all this information via the Integrated Genome Viewer (IGV) [26] to drive the assembly of a panel of 876 pre-designed amplicon-based Illumina TREx assays, selected to specifically target 512 human kinases, according to the schema depicted in Fig. 1. Briefly, in the panel design we selected a first assay for each kinase (ASSAY IN), targeting the kinase domain common to most of the reported kinase isoforms, thus prioritizing the expressed druggable portion of the target sequences (Fig. 1a). Only for CDK3 and TNNI3K no pre-designed TREx assays were available within their respective kinase domains, so they were excluded from the panel. A second assay (ASSAY OUT) was then selected at the maximum sequence distance from ASSAY IN, targeting a region outside the kinase domain and covering the same isoforms encompassed by ASSAY IN (Fig. 1b). An ASSAY OUT fulfilling these criteria could be initially identified for 274 kinases. For other 45 kinases, it was not possible to cover all the isoforms targeted by ASSAY IN with a unique ASSAY OUT: for these, the ASSAY IN was reselected based on a restricted number of isoforms, in order to allow the selection of an ASSAY OUT according to the above criteria. In these latter cases, the initial assay encompassing the kinase domain covering the maximum number of isoforms was retained (ASSAY ADD) in addition to the restricted ASSAY IN and respective ASSAY OUT (Fig. 1b). In this way, we could balance isoform coverage between ASSAY IN and ASSAY OUT without penalizing the number of isoforms detectable for gene expression (ASSAY ADD). In total, an ASSAY OUT could be included for 319 kinases.

This experimental design enables a more robust evaluation of gene expression for those kinases that are probed with more than one independent assay; at the same time, the detection of uneven expression of kinases through the ASSAY IN and the corresponding ASSAY OUT levels can be exploited as readout to suggest the presence of potential gene rearrangements.

### Evaluation of KING-REX performance in detecting kinase gene expression

We evaluated the performance of KING-REX in gene expression quantification by sequencing a panel of 10 colorectal cancer (CRC) cell lines, using KING-REX and whole transcriptome approaches. The same bioinformatics data analysis pipeline was used for both datasets as described in M&M. We calculated the R squared correlation between kinase expression levels measured in the two different protocols in all the analyzed cell lines and observed an average R^2^ > 0.8 of KING-REX with whole transcriptome measurements.

To test the reproducibility of KING-REX, we processed the UHRR control sample in 6 different experiments and the KM12 cell line in 4 different experiments. The R^2^ correlation was calculated in both cases, obtaining an average R^2^ = 0.983 for UHRR and R^2^ = 0.976 for KM12, respectively.

We then evaluated recall, precision and F-measure metrics of KING-REX in detecting kinase signals with varying gene expression level thresholds, considering in-house whole transcriptome data on the same CRC cell lines as reference, as detailed in M&M. Recall and precision values ranged from over 80 to 96%, regardless of the selected threshold (Table 1). The same analysis was also performed using Cancer Cell Line Encyclopedia (CCLE) RNAseq data as reference [27], obtaining comparable results (Table 1).

**Table 1:** Recall, precision and F-measure of KING-REX on a panel of CRC cell lines

Threshold	KING-REX vs. In-house transcriptome data			KING-REX vs. CCLE transcriptome data		
	Recall	Precision	Fmeasure	Recall	Precision	Fmeasure
0.5	92.7	92.9	89.9	90.0	96.5	90.5
1	92.5	93.8	90.1	90.3	96.3	90.5
2	92.5	93.7	89.6	90.8	94.6	89.3
3	91.0	95.4	89.1	90.7	95.2	89.0
4	90.9	95.6	88.7	89.3	95.5	87.5
5	89.2	94.0	85.8	88.7	94.9	86.9
6	86.7	92.6	83.0	86.4	94.0	84.4
7	85.7	87.4	78.2	81.5	88.9	77.2

Recall, precision and F-measure of KING-REX vs. in-house or published CCLE [27] transcriptome data on a panel of colorectal cancer cell lines, with varying detection thresholds. Threshold is expressed as log2 of the normalized counts

Next, we extended the comparative analysis to a more heterogeneous sample panel by selecting cell lines of different tissue origins (BT-474, breast; HPAC, pancreas; K-562, leukemia; KARPAS 299, lymphoma; NCI-H716, large intestine; SNU-1079, biliary tract; U-118 MG, nervous system) for KING-REX analysis and using publicly available CCLE whole transcriptome RNAseq data as reference [27]. Recall, precision and F-measure values were again comparable to the ones observed with the CRC cell line panel (Table 2).

**Table 2:** Recall, precision and F-measure of KING-REX on a heterogeneous panel of cancer cell lines

Treshold	KING-REX vs. CCLE transcriptome data		
	Recall	Precision	Fmeasure
0.5	90.1	97.2	91.8
1	89.4	96.1	90.5
2	89.3	95.5	89.1
3	89.0	94.8	88.0
4	87.0	94.4	85.5
5	86.8	94.0	84.9
6	84.6	93.5	83.2
7	80.4	92.1	78.5

Recall, precision and F-measure of KING-REX vs. CCLE transcriptome data calculated on a heterogeneous panel of cancer cell lines, with varying detection thresholds. Threshold is expressed as log2 of the normalized counts

### Evaluation of KING-REX performance in predicting kinase fusion events

**Fig. 2:**
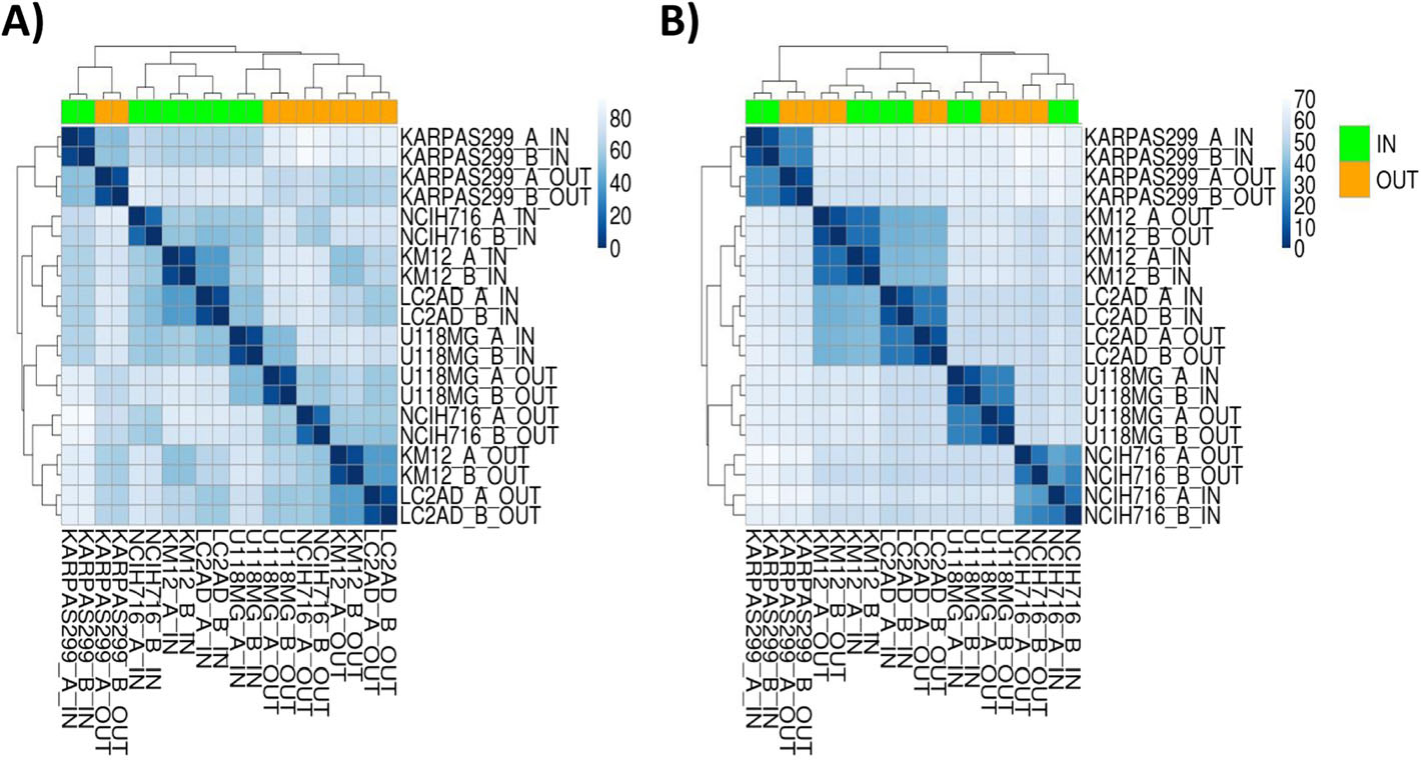
Clustering of cancer cell lines before and after the normalization step. Cluster analysis of a heterogeneous panel of 5 cancer cell lines, tested in duplicate with KING-REX, before (**a**) and after (**b**) the second normalization step in the pipeline for potential kinase fusion event detection. ASSAY_IN expression values for each sample are annotated in green, while ASSAY_OUT in orange. The blue shading indicates the Euclidean distance between the expression values of two samples (cell lines), ranging from dark blue (high similarity) to light blue (low similarity)

In the KING-REX panel assembly, the paired ASSAY IN and corresponding ASSAY OUT, located in opposite 5′ and 3′ transcript ends, support the identification of potential truncated isoforms or gene rearrangements for 319 kinases. For this specific purpose, we have implemented a dedicated pipeline evaluating imbalances between the kinase ASSAY IN and ASSAY OUT signals. We tested the ability of KING-REX to identify imbalanced 5′ vs. 3′ signals in five human cancer cell lines, harboring well known kinase gene fusions: KARPAS 299, KM-12, LC-2/ad, U-118 MG and NCI-H716. KARPAS 299 is a T-cell lymphoma cell line carrying the NPM-ALK gene fusion [28]; KM-12 is a colorectal cancer cell line we had previously reported to express the chimeric TPM3-TRKA protein [29]; LC-2/ad is a lung adenocarcinoma cell line harboring a CCD6-RET fusion [30]; U-118 MG is a glioblastoma cell line characterized by the presence of a FIG(GOPC)-ROS1 rearrangement [31]. NCI-H716 colorectal cancer cell line was included as a control, harboring an amplified full length FGFR2 kinase, whose hyper-activation is due to gene amplification and not to the presence of a concomitant fusion at the FGFR2 C-terminal with COL14A1 gene, conserving an intact kinase sequence [32]. All these cell lines were sequenced in duplicate using KING-REX and analyzed with the kinase fusion event detection pipeline using ASSAY IN and ASSAY OUT measurements (Fig. 2). In the pipeline, after the standard data normalization step (Fig. 2a), results are further processed with a second normalization step, introduced to balance for possible technical differences between ASSAY IN and ASSAY OUT signals, related to primer efficiency, differential end degradation and/or RNA reverse transcription performance. This resulted in the expected clustering of IN and OUT assays belonging to the same cell line (Fig. 2b).

Imbalanced ASSAY IN and ASSAY OUT signals in all the kinases involved in known gene rearrangements were clearly detected by differential gene expression analysis (Table 3). Gene fusions were confirmed by RT-qPCR (Additional file 1: Figure S1). No imbalanced signals could be detected in the negative control cell line NCI-H716, harboring a nearly full length FGFR2 kinase, covered by both ASSAY IN and OUT for FGFR2 (Table 3).

**Table 3:** Prediction of kinase fusion events

Sample	Kinase	FC	*P*-value	IN_A	IN_B	OUT_A	OUT_B
KARPAS 299	ALK	15.83	2.00E-308	12.8	12.9	0.0	0.0
KM-12	NTRK1	10.58	3.94E-236	12.4	12.1	2.3	1.6
LC-2/ad	RET	10.79	6.23E-219	11.8	11.7	1.6	1.6
U-118 MG	ROS1	13.85	4.58E-174	10.9	10.7	0.0	0.0
NCI-H716	FGFR2	–	–				

Imbalanced kinases detected by KING-REX analysis in a panel of cancer cell lines harboring known kinase gene fusions. (*FC* = Log2 fold change between ASSAY IN and ASSAY OUT; *IN* = ASSAY IN expression value for duplicates A and B; *OUT* = ASSAY OUT expression value for duplicates A and B). For differential expression below EdgeR p-value default threshold, no data are reported in the table

### KING-REX limits of detection in gene expression

We explored the limits of detection of KING-REX, both in terms of gene expression measurement and of gene fusion prediction, using 7 samples derived from KARPAS 299 and U-118 MG, both harboring a kinase gene fusion (NPM-ALK in KARPAS 299 and FIG(GOPC)-ROS1 in U-118 MG). We mixed the RNA from the two cell lines in different proportions: 100–0%, 87.5–12.5%; 75–25%, 50–50%, 25–75%, 12.5–87.5%, 0–100%, to simulate different tissue heterogeneity levels as found in clinical cancer samples. Duplicate samples for each mix were then subjected to KING-REX analysis; sequencing results for technical duplicates clearly clustered according to the relative dilution proportions (Additional file 2: Figure S2).

2We next focused on kinases expressed exclusively in U-118 MG or KARPAS 299, i.e. not detected in the other cell line (average gene expression value < 1), and evaluated the disappearance of gene expression signals with the variable increments of KARPAS 299 or U-118MG background, respectively. A clear signal could be observed after incremental dilutions for all the U-118 MG or KARPAS 299 unique kinases, down to the lowest tested concentration (12.5%), regardless of their basal gene expression level (Additional file 3: Table S1).

**Fig. 3:**
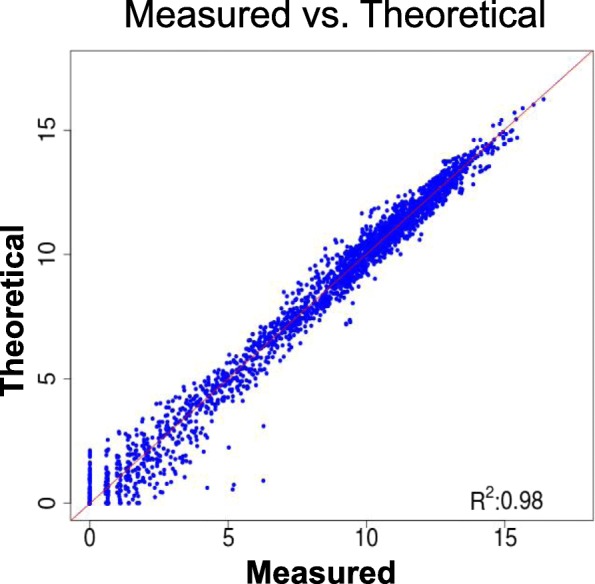
Comparison between measured and theoretical kinase gene expression values. Kinase gene expression values measured with KING-REX within a serially diluted sample set of KARPAS 299 and U-118 MG RNAs, mixed in different proportions (87.5–12.5%; 75–25%, 50–50%, 25–75%, 12.5–87.5%,), plotted vs. ‘theoretical’ expression values, calculated for each kinase and for each dilution factor as described in M&M; the respective correlation R^2^ value is displayed

To evaluate the linearity of kinase gene expression variation within the serially diluted sample set, we compared the measured expression values for each kinase in all the mixed samples versus a ‘theoretical’ value calculated for each dilution as described in M&M, inferred starting from the measured levels in the 100% samples. Despite the complexity of the assay panel composition, theoretical and measured kinase gene expression levels showed high concordance at all dilution levels, as demonstrated by the observed linearity of the reported scatter plot, with an R^2^ = 0.98 (Fig. 3), supporting the robustness of the KING-REX kinase expression profiling approach.

### KING-REX limits of detection in gene fusion prediction

**Fig. 4:**
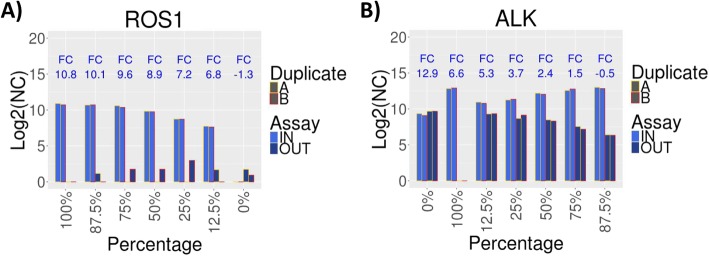
Detection of gene fusion events in diluted samples. **a** KING-REX log_2_(NC) expression values for ROS1 ASSAY IN (light blue) and ASSAY OUT (dark blue) in U-118 MG, diluted in different percentages of KARPAS 299 sample background; **b** KING-REX log_2_(NC) expression values for ALK ASSAY IN (light blue) and ASSAY OUT (dark blue) in KARPAS 299, diluted in different percentages of U-118 MG sample background. The yellow and red bar outline indicates the duplicate A and B of the ASSAY expression value, respectively

We next investigated the limits of detection of fusion events, by evaluating the ability of KING-REX to correctly predict gene fusions in the serially diluted samples described above, based on ASSAY IN and ASSAY OUT imbalances for the rearranged kinases present in the two cell lines. In U-118 MG, the 3′ portion of the ROS1 kinase is detected at high level due to the presence of the FIG-ROS1 gene fusion, while both 5′ and 3′ ROS1 signals are undetectable in KARPAS 299, thus representing an ideal background to determine ROS1 detection limits without confounding factors (Fig. 4a). We observed that the ability of KING-REX to detect the ROS1 5′ vs. 3′ imbalance in a null background is maintained throughout all the dilutions, down to the experimentally tested limit of 12.5% (Table 4).

**Table 4:** Prediction of kinase fusion events at different dilution levels

KARPAS 299 (ALK)	U-118 MG (ROS1)	Kinase	FC	*P*-value	IN_A	IN_B	OUT_A	OUT_B	Class
100.0	0.0	ALK	15.8	2.00E-308	12.8	12.9	0.0	0.0	TP
87.5	12.5	ALK	6.5	0	13.0	12.8	6.4	6.3	TP
87.5	12.5	ROS1	7.4	3.46E-078	7.7	7.6	1.6	0.0	TP
75.0	25.0	ALK	5.3	3.11E-124	12.6	12.8	7.5	7.2	TP
75.0	25.0	ROS1	6.8	1.60E-075	8.7	8.7	0.0	3.0	TP
50.0	50.0	ROS1	9.6	3.09E-194	9.8	9.8	0.0	1.6	TP
50.0	50.0	ALK	3.7	5.46E-105	12.2	12.1	8.5	8.3	TP
25.0	75.0	ROS1	10.3	6.40E-167	10.6	10.3	0.0	1.6	TP
25.0	75.0	ALK	2.35	6.75E-028	11.25	11.36	8.68	9.13	FN
12.5	87.5	ROS1	11.3	2.49E-152	10.7	10.7	1.0	0.0	TP
12.5	87.5	ALK	1.40	2.17E-015	10.95	10.77	9.31	9.34	FN
0.0	100.0	ROS1	13.9	4.58E-174	10.9	10.7	0.0	0.0	TP

Imbalanced 5′ and 3′ kinase signals detected by KING-REX analysis in different dilution mixtures of KARPAS 299 and U-118 MG cell lines. (*FC* = Log2 fold change between ASSAY IN and ASSAY OUT; *IN* = ASSAY IN expression value for duplicates A and B; *OUT* = ASSAY OUT expression value for duplicates A and B)

KARPAS 299, expressing an ALK gene fusion (NPM-ALK), was diluted in the U-118 MG cell line background, in this case expressing a full length ALK, thus representing a more frequent real-life scenario, simulating the case of a tumor mixed with normal adjacent tissue and/or infiltrating lymphocytes, which might express full length ALK (Fig. 4b). In this case, KING-REX could clearly detect the presence of imbalanced ALK gene expression in KARPAS 299 mixtures above 50% proportion (Table 4).

The described cases are only two of many possible experimental scenarios, indicating that the limit of detection of the system is higher when the fusion gene is expressed at lower level or when the background full length WT kinase is highly expressed. Along with these experimental results, we simulated the ‘theoretical’ lower limits of detection of the system at different expression levels of a kinase gene fusion (GE_1_) in a background with varying expression of the full length kinase (GE_2_), by generating a synthetic dataset with different combinations of expression levels, as detailed in M&M. Results in Table 5 show the minimum percentage of sample containing a kinase gene fusion (‘sample 1’) theoretically required by the KING-REX system to successfully detect a gene fusion event with varying background levels of full length kinase in a contaminating sample (‘sample 2’). This simulated dataset was then compared to the available experimental results to verify the theoretical predictions, at least for the tested conditions.

**Table 5:** Theoretical limit of detection for kinase gene fusion events

Gene expression value of FL kinase in sample 2 (GE_2_)	Gene expression value of kinase gene fusion in sample 1 (GE_1_)								
	GE_1_ = 15	GE_1_ = 14	GE_1_ = 13	GE_1_ = 12	GE_1_ = 11	GE_1_ = 10	GE_1_ = 9	GE_1_ = 8	GE_1_ = 7
GE_1_ + 0	81.25	81.25	81.25	81.25	87.50	87.50	87.50	87.50	93.75
GE_1_–1	62.50	62.50	62.50	62.50	68.75	75.00	75.00	75.00	93.75
GE_1_–2	50.00	50.00	43.75	43.75	50.00	56.25	62.50	68.75	87.50
GE_1_–3	31.25	31.25	31.25^b^	37.50	37.50	37.50	43.75	62.50	81.25
GE_1_–4	18.75	18.75	18.75	25.00	31.25	31.25	37.50	50.00	75.00
GE_1_–5	12.50	12.50	12.50	18.75	18.75	25.00	31.25	43.75	68.75
GE_1_–6	6.25	6.25	12.50	12.50	12.50	18.75	31.25	43.75	68.75
GE_1_–7	6.25	6.25	6.25	12.50	12.50	18.75	25.00	37.50	62.50
GE_1_–8	6.25	6.25	6.25	6.25	12.50	12.50	25.00	37.50	
GE_1_–9	6.25	6.25	6.25	6.25	12.50	12.50	25.00		
GE_1_–10	6.25	6.25	6.25	6.25	6.25	12.50			
GE_1_–11	6.25	6.25	6.25	6.25	6.25^a^				
GE_1_-12	6.25	6.25	6.25	6.25					
GE_1_–13	6.25	6.25	6.25						
GE_1_–14	6.25	6.25							
GE_1_–15	6.25								

‘Theoretical’ limit of detection (expressed as the percentage of sample containing the gene fusion vs. the background full length (FL) sample) for the detection of kinases involved in gene fusions with varying expression levels of the fused kinase domain and of the full length WT kinase (background), calculated using ASSAY IN and ASSAY OUT values as described in M&M. *GE*_*1*_ Gene expression level of the fused kinase in sample1, *GE*_*2*_ Gene expression level of the full length kinase (background) in sample 2

^a^theoretical limit of detection calculated in the same case of ROS1 kinase in U-118 MG (GE_1_ = 11) diluted in KARPAS 299 not expressing ROS1 (GE_2_ = 0)

^b^theoretical limit of detection calculated in the same case of ALK kinase in KARPAS 299 (GE_1_ = 13) diluted in U-118 MG expressing full length ALK (GE_2_ = 10)

Indeed, in the case of FIG-ROS1, expressed in U-118 MG cell line and diluted in a null ROS1 background (KARPAS 299), the simulation showed that a ‘theoretical’ limit of detection of 6.25% might be reached (Table 5, highlighted in bold), i.e. below the lowest experimentally tested 12.5% dilution factor (Table 4). Similarly, in the case of KARPAS 299, expressing the NPM-ALK gene fusion diluted in the ALK full length U-118 MG background, a ‘theoretical’ detection limit of 31.25% might be reached (Table 5, highlighted in bold), i.e. between the experimentally tested 50% (positive for fusion detection) and 25% (negative for fusion detection) dilution factors (Table 4), supporting experimental validation conclusions and suggesting an even higher sensitivity of the system.

### KING-REX performance with degraded RNA

We then evaluated the experimental performance of KING-REX on degraded RNA. We extracted RNA from KM-12 cell line and artificially heat degraded it by treating the RNA in aqueous solution at 90 °C for different times (0, 5, 10, 20, 40, 60 min or 5 h). RNA quality and integrity was evaluated by assessing RIN and DV200% parameters for all samples with an Agilent Bioanalyzer. As expected, RNA quality was inversely proportional to the time of exposure to high temperature (Table 6). Heat degraded samples obtained at four representative time points were selected (time = 0, 10, 20 and 60 min) and subjected to KING-REX library preparation in duplicate, followed by sequencing on Illumina MiSeq.

**Table 6:** KING-REX performance with heat degraded RNA

Time (minutes)	RNA quality		Library preparation and sequencing			Gene expression profiling and fusion detection	
	RIN	DV200% (if RIN < 8)	Replicates	Library Conc (ng/μl)	PF Reads (10^6^)	R^2^ with respect T = 0	*P*VAL
0	7.6	94	A	4.2	2.14	1.00	1.21E-143
			B	6.6	2.00		
10	3	90	A	5.5	1.87	0.99	2.14E-223
			B	6.9	1.87		
20	2.2	69	A	2.8	1.80	0.97	5.29E-082
			B	0.9	0.70		
60	2.6	10	A	0.4	0.04	0.78	NA
			B	0.4	0.03		

RNA parameters, library concentration and number of reads obtained from the sequencing of heat degraded KM-12 RNA samples at different time points. Correlation of kinase gene expression profiles at different time point vs. time = 0 (T = 0) and *p*-value relative to the detection of the TPM3-NTRK1 kinase gene fusion are reported (*PF* = Reads passing filter). The experiment was performed in duplicate

Library performance was evaluated in relation to the RNA degradation level. We observed a trend in the reduction of the library concentration with the decrease in RIN and DV200% parameters. KING-REX sequencing performance appeared acceptable in samples with low RIN but still high DV200% values, since an adequate number of reads was still obtained (PF, passing filter reads in Table 6); sequencing analysis of the 60-min sample, characterized by a DV200% of 10%, indicating extreme degradation, yielded a poor number of PF, in line with Illumina recommendations for the TREx protocol, suggesting to process samples preferably with a DV200% > 30% [33].

In terms of gene fusion detection, an imbalanced ASSAY IN/OUT NTRK1 signal in the KM-12 cell line could be appreciated with significant *p*-value at the early time points (time = 0, 10 and 20 min), while this was not possible in sample duplicates with 60 min exposure to heating conditions, due to the generally poor quality of these highly degraded samples (DV200 < 10%). These results demonstrated that KING-REX analysis can be applied also to partially degraded samples.

## Discussion

High biological relevance in different diseases and druggability make kinases a very attractive class of pharmacological targets. Despite the about 40 kinase inhibitor drugs approved in Oncology [5], and the hundreds of compounds in clinical development [4], much of the kinase therapeutic potential remains untapped. Targeted NGS analysis approaches can exploit selected ‘omics’ observations to enable more rapid, cost-effective and focused molecular research screens or diagnostic approaches and are increasingly used. In general, in the implementation of RNAseq targeted panels for the detection of kinase gene expression and/or gene fusions, a compromise must be reached between optimal assay performance and limitations imposed by small scale approaches, by maximizing either sequence coverage or target number. For the detection of gene fusions, assays spanning all the exons of the gene and all exon-exon boundaries of widely characterized kinase diagnostic targets are available (Archer® FusionPlex® NGS assays [14]; QuantideX® NGS RNA Lung Cancer Kit [34]; Ovation® Fusion Panel Target Enrichment System V2 [35]; Ion AmpliSeq RNA Fusion Lung Cancer Research Panel [15, 17, 36]). In our work, we have described the set-up and the performance of KING-REX, a custom targeted RNA approach suitable for the gene expression screening of a comprehensive human kinome panel on Illumina MiSeq or NextSeq platforms. Our panel was intended to maximize kinome coverage (512 kinases) by minimizing the number of per-kinase assays, while retaining the possibility to infer the presence of gene fusions for a wide number of kinases (319), using paired assays located within and outside the catalytic domain. The application of a similar strategy, based on measuring the imbalanced expression between 5′ and 3′ transcript ends, has been restricted so far to the analysis of a limited number of kinases [16, 17]. We implemented an ad hoc data analysis pipeline to streamline both the gene expression analysis workflow and the detection of kinase imbalances as a readout for potential truncated isoform expression or gene fusion events. This was reached by introducing a scaling factor to balance for possible different performances of IN and OUT assays, possibly deriving from technical artifacts, such as primer efficiency, RNA degradation and/or reverse transcription effects. In our work, KING-REX demonstrated a high detection sensitivity and R squared correlation of 0.8 with whole transcriptome results performed in parallel.

An added value of KING-REX system over transcriptome is its focus on kinases, which allows reaching increased read depth for the evaluation of gene expression and for the estimation of the imbalance between gene portions. In the data presented in our manuscript, the average coverage per gene obtained with KING-REX and transcriptome was comparable (about 2000 reads per gene). However, the average coverage per base was over 700 for KING-REX and about 200 for transcriptome analysis, respectively. This allows a higher confidence in estimating the 3′/5′ imbalances used to predict kinase rearrangements; in order to achieve the same coverage per base with a transcriptome analysis, a minimum of 180 M reads per sample should be achieved.

We also extended the analysis to show that KING-REX gene expression detection accuracy was maintained even in heterogeneous RNA mixtures, mimicking the condition of tumor clinical samples, where contamination with adjacent normal/stromal tissue or inflammatory infiltration represents a common scenario. In addition, using cell line models harboring known gene fusions, we have shown that kinase rearrangements can be correctly and robustly detected by the system, distinct from full length sequences, even in complex background mixtures or in heat-degraded RNA, based on the evaluation of the imbalanced measured expression ratio for paired kinase assays.

## Conclusions

To our knowledge, KING-REX is currently the largest targeted approach for expression analysis of kinases which could be used as a rapid and cost effective investigation tool in cancer biology. It represents a useful setup for the comprehensive analysis of the kinome in cancer or other diseases, for applications in the field of the identification of novel, putative kinase targets.

## Methods

### KING-REX panel design

The KING-REX panel was assembled by selecting 876 pre-designed amplicon-based assays from the Illumina DesignStudio Custom Assay Design Tool for use with the TruSeq Targeted RNA Expression (TREx) kit (Illumina, San Diego, CA, USA). Genomics coordinates of all the protein kinases and respective kinase isoforms from UCSC database ([37], assembly hg19 and table knownGene), together with coordinates of the kinase catalytic domains from Superfamily database ([25], superfamily ID number 56112, hg19 genome version) were used to select the assays for the kinome, as described in Results and Fig. 1. In particular, 193 kinases are covered by an ASSAY IN, 274 kinases by an ASSAY IN and an ASSAY OUT, and 45 kinases by an ASSAY IN, an ASSAY OUT and an ASSAY ADD. See Additional file 4: Table S2 for more details.

### Cell cultures and RNA preparation

Human cancer cell lines were maintained as recommended by the suppliers (BT-474, COLO 205, HCT 116, HCT-15, HPAC, NCI-H716, RKO and U-118 MG from ATCC; COLO-678, KARPAS-299, SW 480, SW 948 from ECACC; K-562 and LC-2/ad from DSMZ; LS-180 and SW1417 from ICLC; SNU-1079 from KCLB; KM-12 from NCI). The identity of all cell lines was verified by STR analysis as described in [38]. RNA was extracted from cancer cell line pellets using the RNeasy Mini Kit (Qiagen, Venlo, Netherlands). RNA from KM-12 cell line was heated at 90 °C for 0, 5, 10, 20, 40, 60 min or 5 h in a Hybex thermo-block (SciGene) and immediately chilled and stored at − 20 °C. RNA quality was evaluated by measuring RIN (RNA Integrity Number) and DV200% (% Distribution value of fragments ≥200 nucleotides) parameters using Bioanalyzer 2100 System (Agilent Technologies). Stratagene’s Universal Human Reference RNA (UHRR – Agilent Technologies), an RNA mixture from 10 human cancer cell lines, was used as control in all experiments.

### Library preparation and sequencing

KING-REX library preparation was performed according to the manufacturer’s protocol [33]. Libraries were sequenced in single-end on a MiSeq platform (Illumina, San Diego, CA, USA). Whole transcriptome sequencing library preparation was performed using the TruSeq RNA Access Library Kit (Illumina, San Diego, CA, USA) according to the manufacturer’s protocol and sequenced on a HiSeq1000 (Illumina, San Diego, CA, USA).

### Gene expression analysis

Gene expression quantification from KING-REX and from whole transcriptome data were performed using the same pipeline. Fastq files were aligned to the human reference genome (hg19) using STAR (v. 2.5.1b) [39]. Raw Count (RC) quantification was performed using RSEM tool (v. 1.2.30) [40]. Normalization was performed using DESeq2 (v. 1.12.4) [41] with default parameters and log2 transformed. Gene expression levels were reported as Log_2_ of Normalized Count (NC) for each kinase.

### Pipeline for potential kinase fusion event detection

Raw Count (RC) quantification was performed independently for ASSAY IN and ASSAY OUT using Bedtools Coverage tool (v. 2.22.0) [42]. A first normalization was performed using DESeq2 (v. 1.12.4) [41] with default parameters. A further normalization step was applied to balance possible ASSAY IN and ASSAY OUT expression detection differences due to technical artifacts (i.e. primer efficiency, degradation, RNA reverse transcription effects). In this step, the normalized counts (NC) of ASSAY OUT are corrected with a scaling factor, calculated as the median value of the ratio between ‘ASSAY IN’ NC and the ‘ASSAY OUT’ NC along all samples n:

$$ \mathrm{Scaling}\ \mathrm{factor}=\mathrm{Median}\left({\mathrm{NC}}_{\mathrm{ASSAY}\ \mathrm{INn}}/{\mathrm{NC}}_{\mathrm{ASSAY}\ \mathrm{OUTn}}\right) $$

EdgeR (v. 3.14.0) [43] was applied for the detection of differential expression between the ASSAY IN and ASSAY OUT for each kinase, used as an indicator of potential kinase fusion events.

### Reverse-transcription quantitative PCR

Reverse-Transcription (RT) quantitative PCR (RT-qPCR) was carried out using SYBR green technology; specific primers were designed for the genes of interest using the freely available Primer3 software [44] and synthesized using an Applied Biosystems 3900 Synthesizer. RNA was reverse transcribed into complementary DNA (cDNA) using iScript cDNA Synthesis Kit (Bio-Rad), according to manufacturer’s instructions. Real Time quantitative PCR (qPCR) was carried out on a C1000 Touch ThermalCycler with CFX96 Touch Real-Time PCR Detection System (Bio-Rad), using reagents and materials from Bio-Rad (SsoAdvanced™ Universal SYBR® Green Supermix), according to manufacturer’s instructions, in a volume of 10 μl per reaction, each containing approximately 10–12 ng cDNA, 600 nM primers for 5′ and 3′ regions of the targeted kinases or for the endogenous reference control peptidylprolyl isomerase A (PPIA), as detailed in Additional file 5: Table S3. Each sample was assayed in duplicate qPCR reactions. Quantification of expression levels relative to the UHRR sample was calculated for each targeted sequence following the ΔΔCt method [45]. For comparison with RT-qPCR normalized results, for each IN / OUT assay in each tested cell line, KING-REX Normalized Counts (NC) were processed according to the following formula:

$$ \Delta\ {\mathrm{NC}}_{\mathrm{assay},\mathrm{cell}\ \mathrm{line}}=2\hat{\mkern6mu} \left[\log 2\left({\mathrm{NC}}_{\mathrm{assay},\mathrm{cell}\ \mathrm{line}}\right)-\log 2\left({\mathrm{NC}}_{\mathrm{assay},\mathrm{UHRR}}\right)\right] $$

### Recall, precision and F-measure calculations

Recall, precision and F-measure were evaluated after extracting individual kinase raw counts for each cell line from: i) KING-REX data; ii) in-house transcriptome data, and iii) Cancer Cell Line Encyclopedia (CCLE; [27]) transcriptome data.

Data were normalized using Upper Quartile Normalization (setting the 75th percentile to 1000) [46]. For each kinase in each cell line (cl), the presence (P) or absence (N) of the kinase in the reference data was calculated with variable thresholds (thrs), ranging from 0.5 to 7:

$$ {\displaystyle \begin{array}{l}{\mathrm{Kinase}}_{\left(\mathrm{reference};\mathrm{cl}\right)}>\mathrm{thrs}=>{\mathrm{P}}_{\left(\mathrm{cl}\right)};\\ {}{\mathrm{Kinase}}_{\left(\mathrm{reference};\mathrm{cl}\right)}\le \mathrm{thrs}=>{\mathrm{N}}_{\left(\mathrm{cl}\right)}.\end{array}} $$

For each kinase in each cell line (cl) the concordance of KING-REX with reference control was calculated as follows:

$$ {\displaystyle \begin{array}{l}{\mathrm{Kinase}}_{\left(\mathrm{reference};\mathrm{cl}\right)}>\mathrm{thrs}\ {\mathrm{and}\ \mathrm{Kinase}}_{\left(\mathrm{KING}\hbox{-} \mathrm{REX};\mathrm{cl}\right)}>\mathrm{thrs}=>{\mathrm{TP}}_{\left(\mathrm{cl}\right)};\\ {}{\mathrm{Kinase}}_{\left(\mathrm{reference};\mathrm{cl}\right)}\le \mathrm{thrs}\ {\mathrm{and}\ \mathrm{Kinase}}_{\left(\mathrm{KING}\hbox{-} \mathrm{REX};\mathrm{cl}\right)}\le \mathrm{thrs}=>{\mathrm{TN}}_{\left(\mathrm{cl}\right)};\\ {}{\mathrm{Kinase}}_{\left(\mathrm{reference};\mathrm{cl}\right)}\le \mathrm{thrs}\ {\mathrm{and}\ \mathrm{Kinase}}_{\left(\mathrm{KING}\hbox{-} \mathrm{REX};\mathrm{cl}\right)}>\mathrm{thrs}=>{\mathrm{FP}}_{\left(\mathrm{cl}\right)};\\ {}{\mathrm{Kinase}}_{\left(\mathrm{reference};\mathrm{cl}\right)}>\mathrm{thrs}\ {\mathrm{and}\ \mathrm{Kinase}}_{\left(\mathrm{KING}\hbox{-} \mathrm{REX};\mathrm{cl}\right)}\le \mathrm{thrs}=>{\mathrm{FN}}_{\left(\mathrm{cl}\right)}.\end{array}} $$

From these data, the total number of true positive (TP), true negative (TN), false positive (FP) and false negative (FN) values in all tested cell lines was calculated for each kinase. Recall, precision and F-measure were calculated for each kinase as follows:

$$ {\displaystyle \begin{array}{c}{\mathrm{Recall}}_{\mathrm{kinase}}=\mathrm{TP}/\mathrm{P};\\ {}{\mathrm{Precision}}_{\mathrm{kinase}}=\mathrm{TP}/\left(\mathrm{TP}+\mathrm{FP}\right);\\ {}\mathrm{F}\hbox{-} {\mathrm{measure}}_{\mathrm{kinase}}=\left(2\mathrm{TP}\right)/\left(2\mathrm{TP}+\mathrm{FP}+\mathrm{FN}\right).\end{array}} $$

Tables 1 and 2 report values for recall, precision and F-measure averaged for all kinases in the panel. For more details see Additional file 6: Table S4, Additional file 7: Table S5 and Additional file 8: Table S6.

### Evaluation of measured vs ‘theoretical’ gene expression variation in serially diluted samples

Seven sample mixtures of RNA from KARPAS 299 and U-118 MG cell lines were prepared with the following proportions: 100–0%, 87.5–12.5%; 75–25%, 50–50%, 25–75%, 12.5–87.5%, 0–100%, and subjected to KING-REX library preparation and sequencing. Gene expression values measured for each kinase in the 100% KARPAS 299 or U-118 MG samples were used to infer the ‘theoretical’ expression values expected in each dilution mix, according to the following formula:

$$ \mathrm{Log}2\left[{{\mathrm{NC}}_{\mathrm{KARPAS}\ 299}}^{\ast}\mathrm{P}+{{\mathrm{NC}}_{\mathrm{U}-118\ \mathrm{MG}}}^{\ast}\left(1-\mathrm{P}\right)\right] $$

where NC is the Normalized Counts for each kinase and P corresponds to the serial dilution factor (0.875, 0.75, 0.5, 0.25, or 0.125, respectively). A scatter plot between the measured and ‘theoretical’ sets of data was generated, and R^2^ correlation coefficient was calculated.

### In silico dilutions for the calculation of fusion detection limits

A ‘theoretical’ dilution matrix was created with virtual kinase gene expression levels (GE), to simulate variable tissue mixture conditions in which cells containing a fusion kinase (sample 1, S1) are diluted, or ‘contaminated’, with variable proportions of cells containing a full length kinase (sample 2, S2).

We assumed that, for fusion kinase genes in S1:

$$ {\displaystyle \begin{array}{l}\mathrm{ASSAY}\_{\mathrm{IN}}_{\mathrm{S}1}={\mathrm{GE}}_1\\ {}\mathrm{ASSAY}\_{\mathrm{OUT}}_{\mathrm{S}1}=0\end{array}} $$

where GE_1_ = gene expression of virtual gene fusion in S_1_, ranging from 15 to 7;

and for full length kinase genes in S2:

$$ {\displaystyle \begin{array}{l}\mathrm{ASSAY}\_{\mathrm{IN}}_{\mathrm{S}2}={\mathrm{GE}}_2\\ {}\mathrm{ASSAY}\_{\mathrm{OUT}}_{\mathrm{S}2}={\mathrm{GE}}_2\end{array}} $$

where GE_2_ = gene expression of the full length gene in S_2_ ranging from GE_1_ value to 0. The two artificial S1 and S2 datasets were subjected to KING-REX analysis for potential kinase fusion detection, after generating ‘virtually mixed’ samples with different dilution proportions of S1 and S2, using the following formula:

$$ {\mathrm{Log}}_2\left[{{\mathrm{NC}}_{\mathrm{S}1}}^{\ast}\mathrm{P}+{{\mathrm{NC}}_{\mathrm{S}2}}^{\ast}\left(1-\mathrm{P}\right)\right] $$

Where:

P is the percentage of the sample S_1_ and 1-P is the percentage of sample S_2_; P ranges from 100 to 0% in steps of 6.25%;

NC is the Normalized Counts for each kinase ASSAY IN and ASSAY OUT (including the virtual kinase) obtained with the KING-REX pipeline for potential kinase fusion event detection. For each combination of fixed GE1 and GE2 values and for variable P, we established P_n-1_ as the minimum allowed P, if at P_n_ the gene fusion was not detected by KING-REX pipeline analysis. The matrix in Table 5 shows the minimum allowed P of sample S1, containing the fusion kinase gene, required in a S1/S2 sample mixture for successful gene fusion detection by KING-REX analysis for each combination of virtual GE1 and GE2 values.

## Additional files

Additional file 1: **Figure S1.** Kinase fusion detection by KING-REX vs. RT-qPCR. Relative quantification data as assessed by KING-REX (left) and RT-qPCR (right) analyses for Assay_OUT and Assay_IN regions of ALK in KARPAS299, NTRK1 in KM12, RET in LC2AD and ROS1 in U118MG. Assay_IN and ASSAY_OUT data are reported in blue and red, respectively. Data were normalized as described in M&M section vs. UHRR control sample. (PNG 184 kb)

Additional file 2: **Figure S2.** Distance matrix analysis of KING-REX mixed samples. Distance matrix of KING-REX analysis results for technical duplicates of RNA from two cell lines (KARPAS 299 and U-118 MG), mixed in different percent dilution proportions as indicated on the right side of the graph. The blue shading indicates the Euclidean distance between the expression values of two samples (cell line mixtures), ranging from dark blue (high similarity) to light blue (low similarity). (PNG 220 kb)

Additional file 3: **Table S1.** KING-REX gene expression values of U-118 MG or KARPAS 299 unique kinases in serially diluted cell line RNA mixtures. List of kinases above a mean signal of 5 (Log2NC) in only one of the two tested cell lines (U-118 MG, left panel; or KARPAS 299, right panel) and below a mean signal of 1 (Log2NC) in the other one, with respective gene expression values measured in technical duplicates of RNA mixtures from the two cell lines (KARPAS 299 and U-118 MG) in different proportions (100–0%; 87.5%-12,5%; 75–25%; 50–50%; 25–75%; 12.5–87.5%; 0–100%). The blue shading reflects variations of gene expression values (Log2NC), ranging from 0 (white) to 14 (dark blue). (XLSX 16 kb)

Additional file 4: **Table S2.** Kinase isoforms detected by KING-REX assays. For each kinase, the number of selected assays and the number of isoforms detected by each assay are reported. (XLSX 71 kb)

Additional file 5: **Table S3.** Sequence of primers used in RT-qPCR validation experiments. The sequence of the primers used for the detection of Assay_OUT and Assay_IN portions of ALK, RET, NTRK1, ROS1 and the reference control in RT-qPCR experiment are reported. (XLSX 10 kb)

Additional file 6: **Table S4.** Additional information in support to Table 1, left panel (KING-REX vs. In-house transcriptome data). For each threshold and for each kinase in the KING-REX panel, the following information is reported: (i) the number of cell lines in which the kinase is considered present (P) or absent (N) in the reference dataset; (ii) the total number of true positives (TP), true negatives (TN), false positives (FP) and false negatives (FN); (iii) the recall, precision and F-measure metrics used to obtain data in Table 1, left panel (KING-REX vs. In-house transcriptome data. (XLSX 204 kb)

Additional file 7: **Table S5.** Additional information in support to Table 1, right panel (KING-REX vs. CCLE transcriptome data). For each threshold and for each kinase in the KING-REX panel, the following information is reported: (i) the number of cell lines in which the kinase is considered present (P) or absent (N) in the reference dataset; (ii) the total number of true positives (TP), true negatives (TN), false positives (FP) and false negatives (FN); (iii) the recall, precision and F-measure metrics used to obtain data in Table 1, right panel (KING-REX vs. CCLE transcriptome data). (XLSX 200 kb)

Additional file 8: **Table S6.** Additional information in support to Table 2. For each threshold and for each kinase in the KING-REX panel, the following information is reported: (i) the number of cell lines in which the kinase is considered present (P) or absent (N) in the reference dataset; (ii) the total number of true positives (TP), true negatives (TN), false positives (FP) and false negatives (FN); (iii) the recall, precision and F-measure metrics used to obtain data in Table 2. (XLSX 204 kb)

Additional file 9: KING-REX gene expression data. Normalized counts (Log2transformed) of KING-REX analysis in each sample, as calculated using the pipeline for gene expression analysis described in M&M. (TXT 375 kb)

Additional file 10: Transcriptome Gene expression data. Normalized counts (Log2transformed) of transcriptome analysis in each sample, obtained using the pipeline for gene expression analysis described in M&M. (TXT 3067 kb)

Additional file 11: KING-REX ASSAY IN and ASSAY OUT expression data. KING-REX Normalized counts (Log2transformed) for each ASSAY IN and ASSAY OUT for the 319 kinases in each analyzed sample, obtained using the pipeline for kinase fusion event detection described in M&M. (TXT 460 kb)

## Abbreviations

ATCC: American Type Culture Collection
CCLE: Cancer Cell Line Encyclopedia
CRC: Colorectal cell lines
DSMZ: Deutsche Sammlung von Mikroorganismen und Zellkulturen GmbH
DV200%: % Distrubution value of fragment ≥200 nucleotides
FC: Fold change
FN: False negative
FP: False positive
ICLC: Interlab Cell Line Collection
IGV: Integrated Genome Viewer
KCLB: Korean Cell Line Bank
KING-REX: KINase Gene RNA Expression
NCI: National Cancer Institute
NGS: Next Generation Sequencing
PF: Passing filter reads
RC: Raw counts
NC: Normalized counts
RIN: RNA Integrity Number
STR: Short tandem repeat
TN: True negative
TP: True positive
TREx: Targeted RNA expression

## References

[1] Stransky N (2014). The landscape of kinase fusions in cancer. Nat Commun.

[2] Zhang J (2009). Targeting cancer with small molecule kinase inhibitors. Nat Rev Cancer.

[3] Bhullar KS (2018). Kinase-targeted cancer therapies: progress, challenges and future directions. Mol Cancer.

[4] Klaeger S (2017). The target landscape of clinical kinase drugs. Science.

[5] Ferguson FM, Gray NS. Kinase inhibitors: the road ahead. Nat Rev Drug Discov. 2018. 10.1038/nrd.2018.21. 10.1038/nrd.2018.21 29545548

[6] Manning G (2002). The protein kinase complement of the human genome. Science.

[7] Jabbour E (2013). Tyrosine kinase inhibition: a therapeutic target for the management of chronic-phase chronic myeloid leukemia. Expert Rev Anticancer Ther.

[8] Shaw AT (2013). Crizotinib versus chemotherapy in advanced ALK-positive lung cancer. New Engl J Med.

[9] Shaw AT (2014). Ceritinib in ALK-rearranged non-small-cell lung cancer. New Engl J Med.

[10] Kilpinen S (2010). Analysis of kinase gene expression patterns across 5681 human tissue samples reveals functional genomic taxonomy of the kinome. PLoS One.

[11] Ursu O (2017). Network modeling of kinase inhibitor polypharmacology reveals pathways targeted in chemical screens. PLoS One.

[12] TruSight RNA Pan-Cancer Panel | Cancer gene fusions and expression. https://emea.illumina.com/products/by-type/clinical-research-products/trusight-rna-pan-cancer.html?langsel=/it/. Accessed 20 July 2018.

[13] TruSight RNA Fusion Panel | Fusion detection in cancer research samples. https://emea.illumina.com/products/by-type/clinical-research-products/trusight-rna-fusion.html?langsel=/it/. Accessed 20 July 2018.

[14] FusionPlex Assays | ArcherDX. http://archerdx.com/fusionplex-assays/. Accessed 20 July 2018.

[15] Reeser JW (2017). Validation of a Targeted RNA sequencing assay for kinase fusion detection in solid tumors. J Mol Diagn.

[16] Beadling C (2016). A multiplexed amplicon approach for detecting gene fusions by next-generation sequencing. J Mol Diagn.

[17] Rogers TM (2017). Multiplexed transcriptome analysis to detect ALK, ROS and RET rearrangements in lung cancer. Sci Rep.

[18] TruSeq Targeted RNA Expression Kits | Customizable gene expression studies. https://www.illumina.com/products/by-type/sequencing-kits/library-prep-kits/truseq-targeted-rna.html. Accessed 20 July 2018.

[19] KinBase: Kinase Database at Manning’s Group. http://kinase.com/web/current/kinbase/. Accessed 20 July 2018.

[20] Kinweb: kinase database. https://www.itb.cnr.it/kinweb/. Accessed 20 July 2018.

[21] UniProt. https://www.uniprot.org/docs/pkinfam. Accessed 20 July 2018.

[22] Kinomer. http://www.compbio.dundee.ac.uk/kinomer/bin/kinomes.pl. Accessed 20 July 2018.

[23] KinG, Kinases ecncoded in Genomes. http://king.mbu.iisc.ernet.in/. Accessed 20 July 2018.

[24] Zhao S (2015). A comprehensive evaluation of ensembl, RefSeq, and UCSC annotations in the context of RNA-seq read mapping and gene quantification. BMC Genomics.

[25] Superfamily. http://supfam.org/SUPERFAMILY. Accessed 20 July 2018.

[26] Thorvaldsdóttir H (2013). Integrative genomics viewer (IGV): high-performance genomics data visualization and exploration. Brief Bioinform.

[27] Barretina J (2012). The Cancer cell line encyclopedia enables predictive modelling of anticancer drug sensitivity. Nature.

[28] Hübinger G (1999). The tyrosine kinase NPM-ALK, associated with anaplastic large cell lymphoma, binds the intracellular domain of the surface receptor CD30 but is not activated by CD30 stimulation. Exp Hematol.

[29] Ardini E (2016). Entrectinib, a pan-TRK, ROS1, and ALK inhibitor with activity in multiple molecularly defined Cancer indications. Mol Cancer Ther.

[30] Matsubara D (2012). Identification of CCDC6-RET fusion in the human lung adenocarcinoma cell line, LC-2/ad. J Thorac Oncol.

[31] Kurtis DD, Robert CD (2013). Molecular pathways - ROS1 fusion proteins in cancer. Clin Cancer Res.

[32] Nicorici D (2014). Novel FGFR2 fusion genes in NCI-H716 colorectal cancer cell line. Figshare.

[33] TruSeq® Targeted RNA Expression Reference Guide. https://support.illumina.com/content/dam/illumina-support/documents/documentation/chemistry_documentation/samplepreps_truseq/truseqtargetedrna/truseq-targeted-rna-expression-reference-guide-15034665-01.pdf Accessed 24 July 2018.

[34] QuantideX® NGS RNA Lung Cancer Kit. https://asuragen.com/portfolio/oncology/quantidex-ngs-rna-lung-cancer-kit/. Accessed 20 July 2018.

[35] Ovation® Fusion Panel Target Enrichment System V2 | NuGEN. https://www.nugen.com/products/ovation-fusion-panel-target-enrichment-system-v2. Accessed 20 July 2018.

[36] Simple, Sensitive RNA Fusion Detection With Only 10 ng FFPE RNA | Thermo Fisher Scientific – IT. https://www.thermofisher.com/it/en/home/life-science/cancer-research/cancer-genomics/targeted-sequencing-cancer-mutation-detection/rna-fusion-detection.html. Accessed 20 July 2018.

[37] UCSC Table Browser. https://genome.ucsc.edu/cgi-bin/hgTables. Accessed 20 July 2018.

[38] Somaschini A (2013). Cell line identity finding by fingerprinting, an optimized resource for short tandem repeat profile authentication. Genet Test Mol Biomarkers.

[39] Dobin A (2013). STAR: ultrafast universal RNA-seq aligner. Bioinformatics.

[40] Li B, Dewey CN (2011). RSEM: accurate transcript quantification from RNA-Seq data with or without a reference genome. BMC Bioinformatics.

[41] Love MI (2014). Moderated estimation of fold change and dispersion for RNA-seq data with DESeq2. Genome Biol.

[42] Aaron RQ, Ira MH (2010). BEDTools: a flexible suite of utilities for comparing genomic features. Bioinformatics.

[43] Robinson MD (2010). edgeR: a Bioconductor package for differential expression analysis of digital gene expression data. Bioinformatics.

[44] Primer3. http://biotools.umassmed.edu/bioapps/primer3_www.cgi. Accessed 6 Feb 2019.

[45] Livak KJ (2001). Analysis of relative gene expression data using real-time quantitative PCR and the 2(−Delta DeltaC(T)) method. Methods.

[46] Bullard JH (2010). Evaluation of statistical methods for normalization and differential expression in mRNA-Seq experiments. BMC Bioinformatics.

