# Supplementary figures and images for: Comprehensive kinome NGS targeted expression profiling by KING-REX
Source: BMC Genomics. 2019 Apr 23;20:307. doi: 10.1186/s12864-019-5676-3 (PMC6480677; doi:10.1186/s12864-019-5676-3)

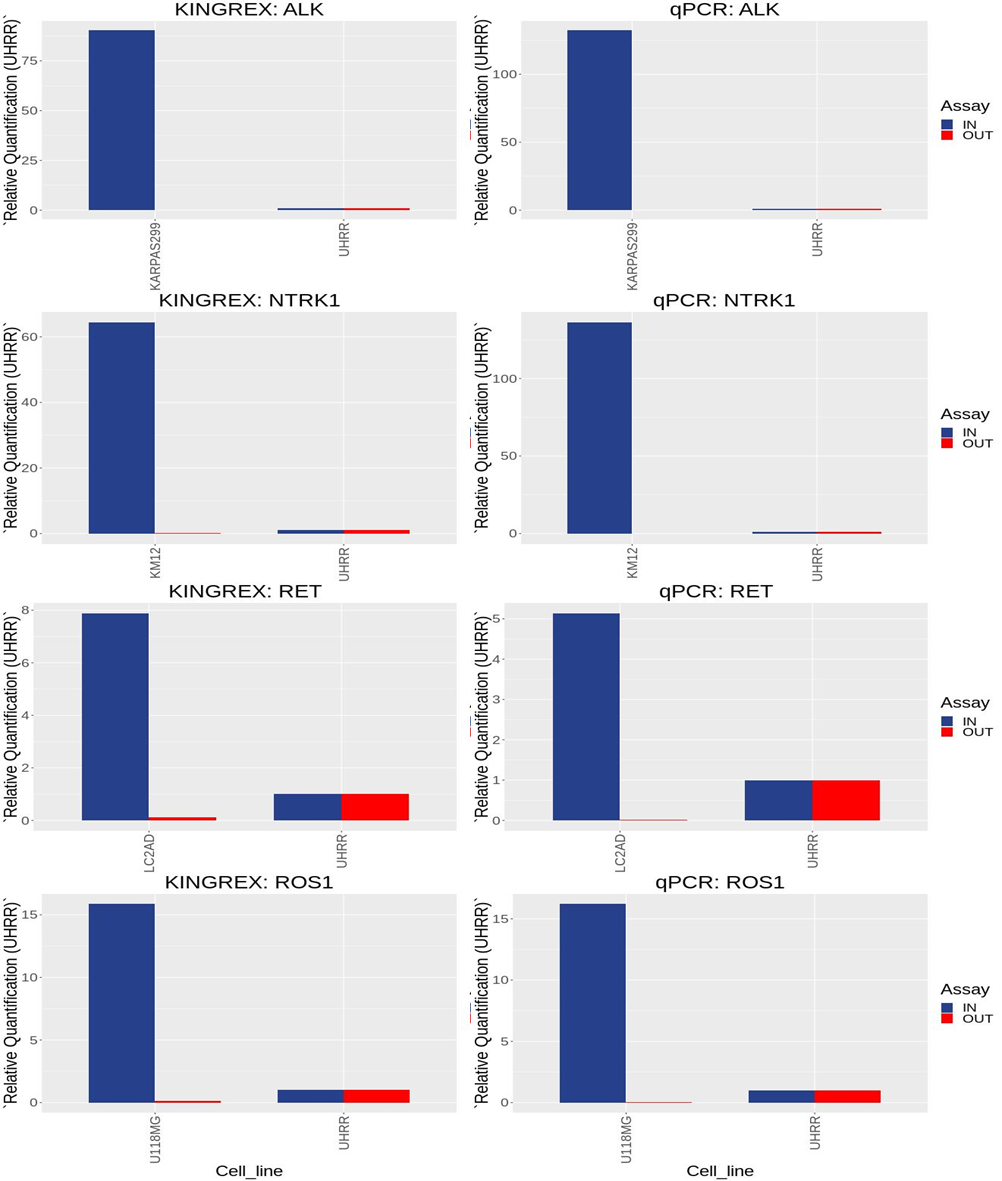

Supplement: Supplementary file 1 — Figure S1. Kinase fusion detection by KING-REX vs. RT-qPCR. Relative quantification data as assessed by KING-REX (left) and RT-qPCR (right) analyses for Assay_OUT and Assay_IN regions of ALK in KARPAS299, NTRK1 in KM12, RET in LC2AD and ROS1 in U118MG. Assay_IN and ASSAY_OUT data are reported in blue and red, respectively. Data were normalized as described in M&M section vs. UHRR control sample. (PNG 184 kb) [file 12864_2019_5676_MOESM1_ESM.png]

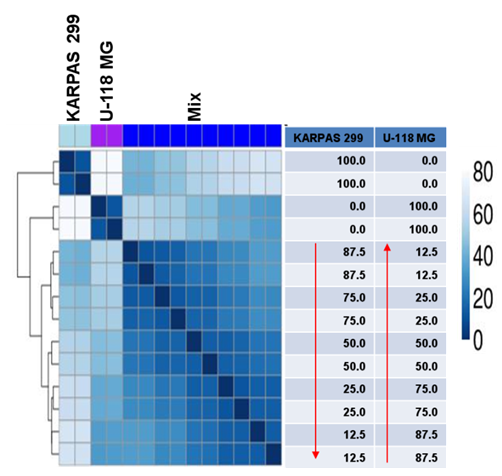

Supplement: Supplementary file 2 — Figure S2. Distance matrix analysis of KING-REX mixed samples. Distance matrix of KING-REX analysis results for technical duplicates of RNA from two cell lines (KARPAS 299 and U-118 MG), mixed in different percent dilution proportions as indicated on the right side of the graph. The blue shading indicates the Euclidean distance between the expression values of two samples (cell line mixtures), ranging from dark blue (high similarity) to light blue (low similarity). (PNG 220 kb) [file 12864_2019_5676_MOESM2_ESM.png]
